# The relationship between *P16*^*INK4A*^ and *TP53* promoter methylation and the risk and prognosis in patients with oesophageal cancer in Thailand

**DOI:** 10.1038/s41598-022-14658-0

**Published:** 2022-06-20

**Authors:** Arisara Poosari, Thitima Nutravong, Wises Namwat, Wiphawan Wasenang, Prakasit Sa-ngiamwibool, Piti Ungareewittaya

**Affiliations:** 1 Department of Microbiology, Faculty of Medicine, Khon Kaen University, Khon Kaen, 40002 Thailand; 2 Faculty of Medical Technology, Nakhon Ratchasima University, Nakhon Ratchasima, 30000 Thailand; 3 Department of Pathology, Faculty of Medicine, Khon Kaen Universtity, Khon Kaen, 40002 Thailand

**Keywords:** Cancer, Microbiology, Gastroenterology, Medical research, Molecular medicine, Risk factors

## Abstract

DNA methylation can regulate the expression of tumour suppressor genes *P16* and *TP53*, environmental factors, which are both important factors related to an increased risk and prognosis of oesophageal cancer (EC). However, the association between these two genes methylation status, as well as the effects of gene-environment interactions, EC risk remains unclear. A Hospital-based case–control study data were collected from 105 new EC cases and 108 controls. Promoter methylation status was investigated for *P16* and *TP53* genes using methylation-specific polymerase (MSP) chain reaction methods with SYBR green. Logistic and Cox regression models were used to analyse the association of *P16* and *TP53* promotor methylation status with EC risk and prognosis, respectively. Our results suggest *P16, TP53* methylation significantly increased the risk of EC (OR = 5.24, 95% CI: 2.57–10.66, *P* < 0.001; OR = 3.38, 95% CI: 1.17–6.67, *P* < 0.001, respectively). In addition, *P16* and *TP53* promoter methylation status and the combined effects between environmental factors and its methylations in tissue were correlated with the EC risk and prognosis of EC patients. As a new biomarker, the methylation of *P16* and *TP53* can serve as a potential predictive biomarker of EC.

## Introduction

Oesophageal cancer (EC) is a multistep process that is the seventh most prevalent cancer globally and the sixth most common cause of cancer-related death worldwide^1^. EC is a chronic disease that affects the upper part of the gastrointestinal tract that is a leading health problem and important cause of death in Thailand. It is highly aggressive and has a poor survival rate^2^. There is an increasing number of new cases reported every year, and the overall estimated age standardised incidence rate, in the eighth rank, is 4.8 and 1.4 cases per 100,000 in males and females, respectively, in 2015^3^. Therefore, the identification of prognostic and predictive biomarkers can provide an index of risk factors for developing EC and help in management of high-risk individuals for prevention and early detection^4^. It is well recognised that the development of EC is a multifactorial induction process, and genetic and epigenetic alterations are involved in it as critical contributors. In several previous studies, much attention has been paid to epigenetic alterations that can serve as reliable indicators of precursor lesions and many types of cancers^5–8^. Epigenetic modification is defined as chromosomal modifications that results in changes to gene expression without alteration of the primary DNA nucleotide sequence^9^. Previous molecular epidemiological studies found that epigenetic modification, as a bridge between genetic and environmental factors is correlated with EC^10,11^. DNA methylation is one of the structures of epigenetic modification, and the expression of both oncogenes and tumour suppressor genes is affected by methylation of DNA^12–14^. Additionally, methylation of DNA, which plays a key role in gene transcription and gene expression, is one of most extensively studied epigenetic alterations. Aberrant methylation of DNA frequently arises at CpG islands within promoter regions, leading to transcription inhibition and gene inactivation. This is recognised as a critical component of the mechanism underlying several tumorigeneses^15–20^. Several studies have demonstrated that the epigenetic mechanism may function as an interaction between environmental risk factors and the genome, and many studies have revealed that lifestyle behaviour and the exposure to environmental factors can affect DNA methylation status and promote tumorigenesis^21–24^.

*P16* (cyclin-dependent kinase inhibitor 2A) is one of the most studied tumour suppressor genes. This gene is located on the human chromosome region 9 of p21. This locus, and especially the *P16* promoter sequence, are CpG-rich regions in which cytosine is usually methylated at the 5′ position by cytosine methyl transferases^25^. The *P16* gene plays essential regulatory roles in the G1 cell cycle pathway, which is related to tumorigenesis when it becomes dysfunctional^26^. Aberrant DNA hypermethylation of promoter regions in the tumour suppressor gene *P16* was reported to be responsible for the silence and inactivation of the corresponding gene involved in carcinogenesis of oesophagus^27^, suggesting that epigenetic alteration in *P16* is involved in the pathogenesis of EC. Numerous studies have revealed that the *P16* gene is frequently found to be methylated in EC^28–30^. A previous study also suggested that aberrant DNA hypermethylation may be a consequence of various environmental, lifestyle-behaviour and dietary factors dependent on the specific region that result in an elevated susceptibility to EC^6,31^. Similarly, a recent study showed that EC related to smoking and drinking involves *P16* silencing resulting from the promoter region hypermethylation^32,33^. These results from previous studies strongly suggested that environmental factors may have an interaction or combination effect with DNA methylation on EC risk. In addition, another study demonstrated that *P16* methylation can be used as an independent prognostic factor at the early stage of EC^34^. The *TP53* gene is an important tumour suppressor gene, which is called protein p53 and is a 53-kD nuclear phosphoprotein (393 amino acids), the product of a 20-Kb gene localised on the short arm of human chromosome 17, at position 17pl3.1^35^. It plays an important role in the cell cycle pathway by controlling cell proliferation^36^. This protein is the principle mediator of cell cycle arrest, senescence and apoptosis in response to DNA damage^37^. Its multiple functions include regulation of gene transcription, leading induction of G1 to S phase arrest and promotion of apoptosis. Recently, the tumour suppresser gene p53 has been considered to be an essential G1 cell cycle regulatory gene whose loss of function is associated with the development of cancer^38^. Epigenetic alteration in this pathway leads to inactivation of these genes, thus leading to cancer. Although a previous study demonstrated that p53 mutation and single-nucleotide polymorphism in codon 72 of the p53 gene are associated with oesophageal carcinogenesis^39^, to the best of our knowledge, few studies have investigated the association between methylation of the *TP53* promoter and EC risk. However, *TP53* promoter methylation in EC is rarely studied and has recently gained more concern. Furthermore, this study identified a prognostic role for promoter methylation of this gene; thus, these markers have yet to be found in their clinical application in EC patients, especially in Thailand. Most previous studies have focused on tissue-derived DNA to investigate the association between gene methylation and cancer risks and prognosis. Moreover, a previous study has reported the alteration of *P16* in EC tissue, but none of the studies investigated both *P16* and *TP53* genes together. Therefore, we carried out this case–control study to investigate the relationship of environmental exposure, *P16* and TP*53* methylation in oesophagus tissue, and their interaction with the incidence of EC. Furthermore, this study also conducted a follow-up study of EC patients to evaluate the association of gene methylation in FFPE tissue with EC prognosis of this patient group in northeastern Thailand.

## Materials and methods

### Study design and population

This study was a hospital-based case–control study with 105 EC cases and 108 cancer-free controls. All subjects were recruited during the same period. All cases and controls were recruited from Srinagarind Hospital, Khon Kaen Province, Northeast Thailand, from 2007 to 2017. A total of 105 newly diagnosed EC patients were included in this study. All cases were histologically confirmed and diagnosed according to the International Classification of Diseases for Oncology (ICD-O 3rd), and the histological diagnosis was reviewed in each case confirmed by pathologists. A medical report was obtained from the pathology department. The control subjects selected were healthy individuals confirmed by physical examination and clinical and biochemical analysis during the same period of case recruitment. Control individuals with a history of malignant tumours or oesophageal malignancy were excluded. Control subjects were randomly sampled from patients undergoing routine endoscopy for investigation of presumed non-malignant conditions, such as gastroesophageal reflux disease. Areas of oesophagus biopsied were macroscopically normal in appearance. All subjects gave written informed consent for their participation in this study. This project was approved by the Human Research and Ethics Committee of Khon Kaen University (Reference No. HE621269).

### Data collection

Data on subjects were obtained with an interviewer-based structured questionnaire, and data were collected from the recruited patients by a face-to-face interview with a trained interviewer using a standardised questionnaire. The questionnaire was considered for content validity by specialists in the field of EC and developed by researchers. All subjects completed a face-to-face investigation questionnaire to obtain demographic characteristic variables and information about social habits and lifestyle. The clinicopathology data were obtained from the electronic medical record system. All cases were followed up until death or the end of the study (31 October, 2019). Overall survival was considered the primary outcome. Thus, the classical endpoint in this study is survival time of EC, and the status of each patient was checked from medical records and by linkage with the death registry of the Thai national statistics database.

### Tissue samples

Tissue samples were obtained from all 105 cases and 108 controls. Specimen tissue was separated into two categories (EC case and control subject) with oesophageal squamous cell carcinoma (ESCC) and oesophageal adenocarcinoma (EAC) defined as the case group, while normal oesophageal tissue representing the control group from 2007 to 2017 were retrieved from paraffin blocks stored in the Department of Pathology, Faculty of Medicine, Khon Kean University. All 213 retrospective formalin-fixed paraffin-embedded (FFPE) samples were collected, including 105 EC and 108 normal tissue samples. These samples were initially diagnosed by the specialist and confirmed by electronic gastroscopy and histopathology reports and diagnosed according to the (ICD-O 3rd).

### Laboratory method

#### DNA extraction and quality control

DNA extraction was from FFPE tissue. The FFPE oesophagus tissue was cut into ten μM sections in a 1.5 microcentrifuge tube. Eight sections from each sample were used for DNA extraction. Prior to sectioning, the microtome and accessories were cleaned with 70% ethanol alcohol. The DNA of the FFPE tissue samples were extracted using a commercially available system, DNeasy blood and tissue kits (Qiagen, Hilden, Germany), according to the manufacturer’s instructions. The yields and quality of the DNA isolated by the kit were measured by use of a Nano Drop ND-1000 Spectrophotometer (Thermo Scientific), and DNA quality was assessed. The housekeeping human beta actin (β-actin) gene served as an endogenous control to guarantee the DNA quality and was detected using a specific primer as previously described^40^. The integrity of the extracted DNA samples was confirmed by amplifying a housekeeping human beta actin (β-actin) gene using SYBR green real-time polymerase chain reaction (PCR). All DNA samples were stored at − 20 ^๐^C for further analyses.

#### Bisulphite modification

Sodium bisulphite modification is based on the selective deamination of unmethylated cytosines to uracil, whereas methylated cytosines remain unchanged. The chemical reaction exhibits specific changes in the DNA sequences that reflect the methylation status of individual cytosine residues. Tissue DNA containing 1 μg was treated with bisulphite using an EZ DNA methylation Gold Kit (Zymo Research Corp., Irvine, CA, USA) according to the manufacturer’s instructions. Briefly, a final volume of 20 µl bisulfited-modified DNA was obtained and used immediately as a template for real-time methylation-specific PCR (MSP) analysis or stored at − 80 °C no longer than 4 weeks.

#### Real-time methylation-specific PCR

The methylation status of *P16* and *TP53* was performed using real-time MSP chain reaction with two primer sets, methylated primer and unmethylated primer. The specific primers of CpG islands related to promoters of these genes were designed^8^ and were checked using Methyl Primer Express software version 1.0 (Applied Biosystems) and Bisearch (http://bisearch.enzim.hu/?m=genompsearch). The methylated primer is specific to fully methylated sequences, while the unmethylated primer is specific to fully unmethylated sequences. PCR amplification and melting curve analysis were performed on an Applied Biosystems 7500 flats system. The PCR reaction mixture was performed in a final volume of 20 μl consisting of 8 ng of bisulphite-modified DNA1XSYBR qPCR Mix (Bio-Rad, Hercules, CA, USA), 2.5 mM MgCl2, 500 µM of each dNTP and 0.3 μM of each forward and reverse primer. The optimal conditions of MSP are shown in Table Supplementary (S1). The amplification steps were as follows: initial denaturation at 94 °C for 5 min, 40 cycles of denaturation at 94 °C for 45 s, annealing at optimal temperature for 40 s and extension at 72 °C for 40 s, and a final extension at 72 °C for 5 min. The melting step was obtained from 60 to 95 °C in duplicate to confirm repeatability, and they were averaged following analysis. Human peripheral blood leukocyte DNA was treated with SssI methyl transferase (New England Biolabs, Inc., Beverly, MA, USA) as fully methylated DNA, while the leukocyte DNA was non-treated as fully unmethylated DNA. Each sample was done in duplicate. The melting temperature (Tm) of fully unmethylated DNA controls with the specific primer sets was defined as the cut-off point for methylation status. MSP assay of *P16* and *TP53* methylation in tissue DNA samples was determined by using fully methylated controls and fully unmethylated DNA controls with individual specific primer set is shown in Figs. S1 and S2. The data shows the amplification curve and melting curve, which represent the cut-off point of Tm for determining methylation status. Thus, these figures present the standard melting curves and melting peaks, for the definition *P16* and *TP53* methylation status.

### Statistical analysis

The demographic characteristics of the subjects were summarised using descriptive statistics. Statistical analysis was performed to examine the differences in characteristics between cases and control group using the chi-squared test for categorical variables, and continuous variables were analysed using t-tests or rank-sum tests. Logistic regression analysis was applied to explore the relation of DNA methylation and environmental factors with EC. Odds ratios (ORs) and 95% confidence intervals (95% CIs) were applied to assess the strength of association between *P16* and *TP53* methylation status along with environmental factors and EC risk derived from unconditional logistic regression. Univariate and multivariate logistic regression analyses were approved to evaluate the relationships between gene methylation, environmental factors and EC risk, and the relationships between gene methylation and environmental factors. The interactions of gene methylation and environmental factors on EC risk were estimated on a multiplicative scale with a product-term coefficient using multivariate logistic regression. The combined effects of gene methylation and environmental factors on EC risk were calculated by crossover analysis. Multivariable logistic regression was used to compute adjusted odds ratios (ORadj.) and 95% confidence intervals (95% CIs) for the association between DNA methylation, environmental factors and EC, while controlling for the effects of confounding variables. The backward stepwise elimination method was used as the model fitting strategy. A likelihood ratio test was performed to assess the goodness-of-fit of the final model. The survival probabilities were determined by the Kaplan–Meier method. The statistic used to compare survival between groups was performed by using the log-rank test. Univariate and multivariate Cox proportional hazard regression models were used to estimate the association between explanatory gene methylation and clinical characteristics and prognosis of EC patients, and the results were presented in the form of crude and adjusted hazard ratios (HRs) and their 95% CIs. HR was assessed to investigate the magnitude and direction of the effect. All statistical analysis was performed using Stata® software (version 13.0), with the test statistics two-sided, and a *p*-value less than 0.05 was considered statistically significant.

### Ethics declarations

This present study was approved by the Khon Kaen University Ethics Committee for Human Research, based on the Declaration of Helsinki and the ICH Good Clinical Practice Guidelines; reference number HE621269.

## Results

### Demographic characteristics of subjects

The study included 105 cases and 108 controls. The characteristics of all subjects are provided in Table 1. The gender and age distribution differed between the cases and controls (men, 60 and 47%, respectively; women, 40 and 53%, respectively). The mean age (± SD) of the cases and controls was 60.4 years (± 8.8) and 60.0 years (± 9.2), respectively. The demographics of the subjects’ lifestyle habits were evaluated. Drinking status, smoking and betel chewing were more prevalent among the cases than the controls. The distribution frequency of a family history of cancer between cases and controls statistically differed (*P* < 0.05). The results showed no difference between cases and controls in terms of body mass index (BMI) and marital status (*P* = 0.681 and *P* = 0.945, respectively).

**Table 1:** Demographic characteristics of the oesophageal cancer cases and controls.

Characteristics	Cases		Controls		*P*-value
	N = 105		N = 108		
**Gender**					
Female	42	(40.0%)	58	(53.7%)	0.053^a^
Male	63	(60.0%)	50	(46.3%)	
Age (mean ± SD)	60.4 ± 8.8		60.0 ± 9.2		0.274^b^
< 60	50	(47.6%)	60	(55.5%)	
≥ 60	55	(52.4%)	48	(44.5%)	
**Drinking status**					
Non-drinker	33	(31.4%)	65	(61.0%)	< 0.001^a^
Drinker	72	(68.6%)	43	(39.0%)	
**Smoking status**					
Non-smoker	37	(35.5%)	74	(68.6%)	< 0.001^a^
Smoker	68	(64.7%)	34	(31.4%)	
**Family history of cancer in first-degree relatives**					
No	27	(25.7%)	62	(57.1%)	< 0.001^a^
Yes	78	(74.3%)	46	(42.9%)	
**Body mass index; BMI (kg/m**^**2**^)** (mean ± SD)**	24.1 ± 3.62		23.4 ± 3.46		0.681^b^
< 23.00	49	(46.7%)	53	(51.4%)	
≥ 23.00	56	(53.3%)	55	(48.6%)	
**Marital status**					0.945^a^
Single	9	(8.6%)	9	(8.3%)	
Married	88	(83.8%)	92	(85.2%)	
Separated	8	(7.6%)	7	(6.5%)	
**Betel chewing**					0.703^a^
Non-betel chewers	88	(88.8%)	93	(86.1%)	
Betel chewers	17	(16.2%)	15	(13.9%)	

^a^*p*; *p*-value differences between cases and controls were detected using the Chi-squared test.

^b^*p*; *p*-value differences between cases and controls were detected using the Wilcoxon rank-sum test.

### Association between environmental factors and EC risk

The correlations of environmental exposure with EC risk are presented in Table S2. The primary outcomes of the multivariable analysis are depicted in this table, and after backward conditional selection analysis, our results showed that alcohol drinking, smoking and family history of cancer significantly increase the risk of EC (*P* < 0.05). BMI, marital status and betel chewing were not significantly associated with EC.

### Association between the methylation status of P16 and TP53 and risk of EC

Table 2 provides the results of gene methylation status relative to EC, which revealed that *P16* methylation had an increased EC risk and was statistically significant (OR_adj_ = 5.24, 95% CI: 2.57–10.66). Furthermore, our study also found that *TP53* methylation was significantly associated with EC at (*P* < 0.001).

**Table 2:** Associations between the methylation status of P16 and TP53 and risk of oesophageal cancer.

Methylation Status	Cases (%)	Control (%)	OR_C_ (95%CI)	p-value	ORadj (95%CI)	*p*-value
***P16 gene***						
Unmethylated	22 (20.9)	68 (62.9)	1.000	0.001	1.000	< 0.001
Methylated	83 (79.1)	40 (37.1)	5.64 (2.59–11.31)		5.24 (2.57–10.66)	
***TP53 gene***						
Unmethylated	28 (26.7)	67 (60.9)	1.000	0.001	1.000	< 0.001
Methylated	77 (73.3)	41 (39.1)	4.09 (2.11–7.69 )		3.38 (1.71–6.67 )	

ORc: crude odds ratio, ORadj.: adjusted odds ratio, 95% CI: 95% confidence interval, *P16*: (cyclin-dependent kinase inhibitor 2A), *TP53*: (tumour protein p53), Adjusted for age, gender, drinking, smoking, family history of cancer, BMI, marital status, betel chewing.

### Relationships between *P16* and *TP53* methylation and environmental factors

The relationship between *P16* and *TP53* methylation and environmental factors were explored in all 105 EC cases and 108 controls. As presented in Tables S3 and S4, drinking status and smoking were associated with increased risk of *P16* methylation (OR_adj_ = 2.49, 95% CI: 1.71–3.13, *P* = 0.029; OR_adj_ = 2.36, 95% CI: 1.36–4.75, *P* = 0.003, respectively). Moreover, the risk of *TP53* methylation was slightly higher in subjects who were drinking alcohol and smoking (an increased OR), and the risk associated with these factors was statistically significant (OR_adj_ = 2.06, 95% CI: 1.71–3.70, *P* = 0.013; OR_adj_ = 1.61, 95% CI: 1.15–3.21, *P* = 0.041, respectively).

### The effect of interactions between *P16* and *TP53* methylation and their interactions with environmental factors on the risk of EC

The results found that the combined effects between *P16* methylation and alcohol drinking and smoking on EC risk existed (OR_c_ = 2.43, 95% CI: 1.41–4.38, *P* = 0.002; OR_c_ = 2.75, 95% CI: 1.73–5.10, *P* = 0.001 respectively), whereas no interaction between *P16* methylation and environmental factors on the EC risk was showed, as seen in Table S5. As for the *TP53* gene, its methylation and some environmental factors have combined effects on the risk of EC (*P* < 0.05) (Table S6). Additionally, the results in Table S7 illustrate that *P16* methylation did interact with *TP53* methylation on EC risk.

### Characteristics of EC patients

Of the 105 subjects who were recruited as EC cases in this study, all EC patients were included in this 10-year follow-up study. The association between demographic clinicopathological and EC prognosis was analysed, as presented in Table S8. Although the correlation between each demographic characteristic and prognosis of EC patients was not statistically significant, age, gender, BMI, family history of cancer, marital status and betel chewing were still used as the adjustment factors in analysing the relationship between clinical characteristics and EC prognosis, and these factors were common confounder factors in this study. Multivariate analysis based on Cox proportional hazard regression revealed that TNM stage, histology grading (poor differentiation) and lymph node metastasis were statistically significantly associated with EC prognosis (*P* < 0.05) (Table 3). The results from backward condition selected suggested that EC patients with TNM stage III had marginally poorer prognoses (HR = 1.25, 95% CI: 1.44- 3.42, *P* = 0.046) and EC patients with stage IV had seriously poorer prognoses (HR = 2.68, 95% CI: 1.04–6.93, *P* = 0.041). In addition, metastasis was also associated with poorer prognosis of EC patients (HR = 1.52, 95% CI: 1.21–2.48, *P* = 0.013) (Table S9).

**Table 3:** Association between clinicopathological factors and EC prognosis.

Variable	Cases (% )	Median survival (Months) (95% CI)	Crude HR (95% CI)	*p*-value	Adjust HR (95% CI)	*p*-value
**Region of cancer (Typical oesophagostomy)**						
Upper site	5 (4.7%)	12.8 (9.23–16.36)	1.00		1.00	
Middle site	73(69.5%)	8.7 (7.13–10.26)	0.54 (0.18–1.59)	0.308	0.27 (0.08–1.18)	0.291
Lower site	27(25.7%)	5.4 (0.40–10.39)	0.58 (0.21–1.62)	0.271	0.31 (0.11–1.78)	0.286
**Histology type**						
Adenocarcinoma	28 (26.7%)	7.3 (3.91–10.69)	1.00	0.785	1.00	0.696
Squamous cell carcinoma	77 (73.3%)	8.8 (5.99–11.61)	1.06 (0.66–1.71)		1.10 (0.67–1.81)	
**Histology grading (Goseki classification)**						
Group I,II (well or moderate differentiation)	33 (31.4%)	12.8 (5.96–19.63)	1.00	0.637	1.00	0.048
Group III,IV (poor differentiation)	72 (68.6%)	8.7 (6.13–11.26)	1.15 (0.69–1.92)		1.22 (1.09–2.82)	
**Stage of disease (TNM classification)**						
Stage I (IA,IB)	10 (9.5%)	39.9 (3.32–74.88)	1.00		1.00	
Stage II	17 (16.2%)	20.7 (7.57–33.83)	1.31 (0.49–3.44)	0.589	0.74 (0.24–3.01)	0.604
Stage III (IIIA , IIIB)	21 (22.9%)	8.6 (7.26–9.93)	1.48 (1.57–5.43)	0.038	1.25 (1.44–3.42)	0.046
Stage IV	57 (51.4%)	6.1 (4.42–7.78)	2.71 (1.16–6.36)	0.022	2.68 (1.04–6.93)	0.041
**Metastasis**						
No	35 (33.3%)	12.0 (7.22–16.77)	1.00	0.009	1.00	0.013
Yes	70 (66.7%)	7.1 (3.50–10.69)	1.81 (1.17–2.88)		1.52 (1.21–2.48)	
**Complication**						
No	71 (67.6%)	8.9 (4.81–12.31)	1.00	0.804	1.00	0.875
Yes	34 (32.4%)	8.1 (7.22–16.77)	0.94 (0.61–1.47)		0.96 (0.59–1.56)	

Oesophageal cancer (95% CI): 95% confidence interval, HR hazard ratio, adjust for age, gender, BMI and comorbidity by using Cox proportional hazards regression models. *p*-value from partial likelihood ratio test.

### Association between *P16* and *TP53* methylation status and EC prognosis

**Figure 1:**
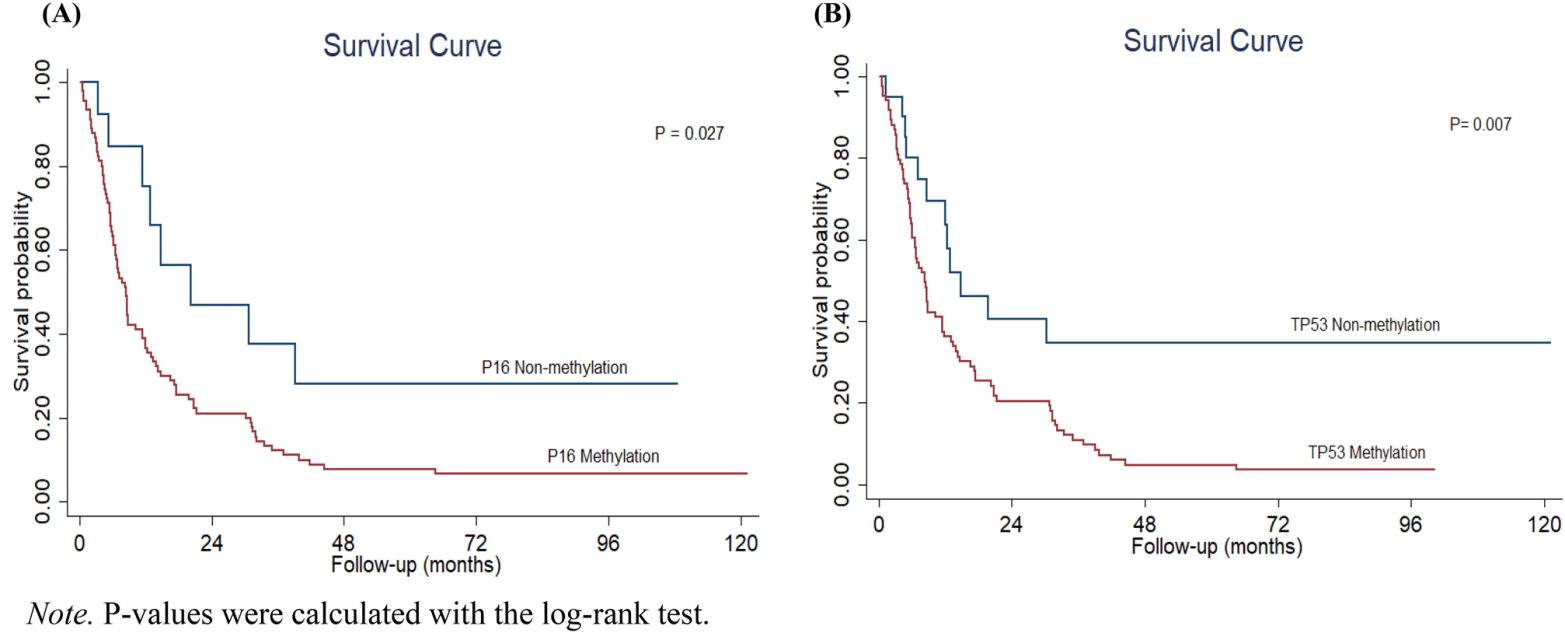
Kaplan–Meier Survival Curves of the Association Between P16 (**A**) and TP53 (**B**) Methylation and EC Prognosis. Note. *P*-values were calculated with the log-rank test.

In this study, the potential impact of *P16* and *TP53* methylation on EC prognosis was investigated. Our data are shown in Table 4. When compared with non-methylation, both *P16* and *TP53* methylation were strongly associated with EC prognosis (HR = 2.82, 95% CI: 1.13–5.06; HR = 2.95, 95% CI: 1.34–5.47, respectively). In Fig. 1, our results show the association between *P16* and *TP53* methylation status and EC patients’ prognosis by the Kaplan–Meier method. Briefly, this study found that DNA methylation of *P16* and *TP53* was associated with patients’ overall survival. In addition, EC patients who had *P16* and *TP53* methylation showed significantly shorter overall survival than those who had non-methylation status (*P* = 0.027; *P* = 0.007, respectively).

**Table 4:** Association between methylation status of P16 and TP53 and EC prognosis.

Variable	Cases (%)	Median survival (Months) (95% CI)	Crude HR (95% CI)	*p*-value	Adjust HR (95% CI)	*p*-value
***P16* Methylation status**						
Unmethylated *P16*	22 (20.9%)	20.2 (4.95–15.34)	1.00	0.013	1.00	0.027
Methylated *P16*	83 (79.1%)	8.5 (6.83–10.17)	2.28 (1.10–4.71)		2.82 (1.13–5.06)	
***TP53* Methylation status**						
Unmethylated *TP53*	28 (26.7%)	14.8 (4.85–24.75)	1.00	0.002	1.00	0.007
Methylated *TP53*	77 (73.3%)	8.4 (6.52–10.28)	2.37 (1.28–4.37)		2.95 (1.34–5.47)	

Oesophageal cancer; (95% CI): 95% confidence interval, HR hazard ratio, *P16*: (cyclin-dependent kinase inhibitor 2A), *TP53*: (tumour protein p53), adjust for age, gender, BMI, histology type, grading, TNM state, metastasis, complication and comorbidity by using Cox proportional hazards regression models. *p-value* from partial likelihood ratio test.

## Discussion

To the key point, this study is the first report on the molecular characteristics of EC from FFPE tissue patients, and this is the first study showing the possible interaction and association of epigenetics with environmental factors and the risk and prognosis in patients with EC in Thailand. EC is the result of both effects of environmental factors and epigenetic susceptibility, and behaviour is one of the most important influencing factors. Moreover, our results found that the *P16* and *TP53* methylation status was significantly associated with poor prognosis in EC patients. In this case–control study, our objective was to investigate the association and interaction of various environmental, behaviour factors and carcinogen metabolising aberrant DNA methylation, how they are related to the incidence of EC and promoter methylation status of frequency methylated tumour suppressor genes *P16, TP53* and clinic pathological characteristics on the survival of EC patients among Thai populations. In this research, we exploited an approach to study complex environmental factors and epigenetics in EC. The aetiology and pathogenesis of EC involve complicated interactions between epigenetic, genetic and environmental factors, and it is a complex progressive disease with multiple stages^6^. The results indicated tobacco smoking and alcohol consumption as the major risk factors for EC and its probable role inducing promoter methylation in the upper gastrointestinal tract. Moreover, the results of this study showed higher *P16* and *TP53* promoter methylation frequency in EC patients than normal controls. In the current study, epigenetic modifications, including DNA methylation, were significantly related to the pathogenesis of EC. Similarly, previous research by Zhou et al. (2017)^41^ reviewed a systematic meta-analysis based on 42 articles (including 2656 ESCC cases and 2979 normal controls). Their results showed a significant increase in the frequency of *CDKN2A/P16* methylation during ESCC carcinogenesis (ESCC vs normal control, OR = 12.60, *P* < *0.01*. The association of DNA methylation and EC is the best model to validate in EC acting as a non-invasive potential biomarker. Although there have been several reports that the frequency of *P16* and *TP53* promoter methylation is higher in ESCC samples than those from cancer-free controls, the association and the role of these genes’ methylation in ESCC remain controversial^8^. However, the correlation of *P16* and *TP53* methylation has not yet been reported, especially in Thailand; therefore, this study can be performed as a new finding that was conducted in a hospital-based case–control study in order to detect aberrant DNA methylation in tissue samples from EC patients using MSP chain reaction. This research incorporated a total 213 participants, including 105 EC cancers and 108 control samples. In this study, we observed the possible association and interaction of epigenetic and environmental factors with the risk of EC. The results indicated that methylation of *P16* and *TP53* was significantly associated with the risk of EC. In addition, alcohol consumption and smoking status may have tumour-promoting effects of aberrant DNA methylation of *P16* and *TP53* genes. This interesting incidence was also consistent with in previous research^10,22^. As mentioned, the consumption of alcohol and tobacco contribute to ESCC carcinogenesis. A previous study found that promoter region hypermethylation was associated with tobacco consumption by analysing a group of tumour suppressor genes in ESCC patients when compared to the control group^42^. Similar results from Ye and Xu^43^ found that Benzo (α) pyrene diol epoxide, as a carcinogen present in tobacco smoke, alcohol drinking and environmental pollution, has been shown to induce aberrant DNA methylation (such as *P16*, *TP53* and *KRAS* genes). Although a previous study also confirmed tobacco smoking as a predominant risk factor for ESCC, the highest risk was associated with tobacco chewing in the concerned population. Tobacco is chewed in various forms either alone or with slaked lime or betel quid, and the spit is often swallowed. Like tobacco smoke, smokeless forms of tobacco are also known to contain several carcinogenic compounds, the most potent of which are the tobacco-specific N-nitrosamines like N’-nitrosonornicotine (NNN) and 4-(methylnitrosamino)- 1-(3-pyridyl)-1-butanone (NNK)^44^. Many epidemiological studies have consistently shown that alcohol drinking and tobacco use have synergistic effects on carcinogenesis, when combined use explained more than 70% of ESCC^45,46^. Indeed, Fan et al.^47^ demonstrated that various factors from exposure to the same carcinogen (i.e., nitrogen, cigarette smoke, alcohol drinking) have been identified as risk factors for EC in a high-risk population, and these modifiable factors should be part of any primary prevention strategy for this EC, which has a very poor prognosis. Although the relationship between EC risk and DNA methylation has been previously reported, evidence indicating the association of environmental factors with DNA methylation and EC risk and prognosis in EC patients remains limited.

The association between methylation status of *P16*, *TP53* and EC prognosis was also explored in this study. These results reported on the effect of DNA methylation and clinicopathological characteristics on the survival of EC patients among the Thai population. As expected, we found that tumour characteristics, such as stage of cancer and metastasis, were associated with survival rates. In addition, some of the interesting factors, such as *P16* and *TP53* methylation, were related to the mortality of EC cancer patients. This finding suggested that DNA methylation is an epigenetic event commonly found in EC. The DNA methylation of these genes, at least in part, may potentially lead to gene silencing, which may have an important impact on cancer cell survival and progression. This finding is also consistent with previous studies^11^. Our results showed that the stage of cancer was the greatest factor affecting the survival of EC, which is similar to several previous studies that have reported this, especially in the advanced stage of the disease^48,49^. Moreover, in our finding, stage IV of EC related to the declining of survival when compared to other stages, as mentioned above; this is consistent with the reported by Fujiwara et al.^50^. A previous study concluded that differences in pathogenesis and tumour biology can be induced given the variance of survival time in EC patients. In addition, stage IV also contains not only those patients with metastasis but also those with advanced primary tumour and region lymph node status without metastasis. Thus, EC patients with stage IV (poor differentiation) have a significantly poor prognosis. Our research showed high methylation levels of *P16* and *TP53* genes, and these genes were associated with EC. Briefly, this finding suggested that DNA methylation is a common form of epigenetic modification, which has a crucial role in human malignancies, such as EC^51^, lung cancer^17^ and gastric cancer^52^. Abnormal methylation in the promoter region of tumour suppressor genes is one of the most common mechanisms of modification, and the results in target gene transcriptional silencing, so that DNA methylation has become a credible potential biomarker for early detection and diagnosis of cancer, and it may have a great impact on cancer cell survival and prognosis^18,19^. The present study showed that promoter methylation of two genes, namely *P16* and *TP53*, were not only tumour suppressor specific, but also correlated with a patient’s overall survival time. An important aspect of this study is the potential methylation status of *P16* and *TP53*, which can serve as predictive biomarkers for EC. *P16* methylation is a favourable predictive marker, and *TP53* methylation is clear to use for predictions; when used in combination, it may strengthen their potential marker. In particular, promoter methylation of *P16* and *TP53* are involved in the progression of EC, and clinical pathology, such as stage of disease and metastasis, is related to poor survival outcomes and shorter survival. Therefore, further large-scale prospective studies are needed to contribute to a deeper and more extensive understanding of epigenetic and environmental factors and their interaction in the different stages of carcinogenesis of EC. There were several limitations in the present study. First, recall bias might be inevitable for gathering information on environmental factors, although we attempted to reduce this bias. In particular, the collection data about the frequency and duration of use of alcohol and smoking are not detailed, and these data could affect the methylation of candidate genes. Second, we cannot determine the possible mechanisms that affect methylation differences in these genes between the cases and control group. This study had important strengths, a case–control study was conducted to apply molecular epidemiology pathology methods to the association and interaction of environmental factors and epigenetic biomarkers with the survival of EC.

## Conclusion

In conclusion, our findings provide insight into aberrant DNA methylation of *P16* and *TP53* as an epigenetic event of EC, and these results indicated that *P16* and *TP53* promoter methylation status and the combined effects between environmental factors and their methylations in tissue were correlated with the EC risk and prognosis of EC patients. The methylation of *P16* and *TP53* can serve as a potential predictive biomarker of EC.

## Supplementary Information

Supplementary Information.

## Data Availability

The datasets during and/or analyzed during the current study available from the corresponding author on reasonable request.

## Abbreviations

°C: Celsius
95% CI: 95% Confidence interval
DNA: Deoxyribonucleic acid
OR adj: Adjusted odds ratios
OR c: Crude odds ratios
HR: Hazard ration
MSP: Methylation-specific polymerase chain reaction
*P16*: Cyclin-dependent kinase inhibitor 2A
*TP53*: Tumour protein p53
