# Supplementary material for: The relationship between P16INK4A and TP53 promoter methylation and the risk and prognosis in patients with oesophageal cancer in Thailand
Source: Sci Rep. 2022 Jun 20;12:10337. doi: 10.1038/s41598-022-14658-0 (PMC9209525; doi:10.1038/s41598-022-14658-0)
Supplement: Supplementary file 1 — Supplementary Information. [file 41598_2022_14658_MOESM1_ESM.docx]

**Additional File 1: Table S1.** *Primer Sequences and Optimal Conditions for Methylation-Specific PCR*

| **Gene** | **Primer** | **Primer Sequences**  **(5’ to 3’)** | **Product Size (bp)** | **Ta (C)** |
| --- | --- | --- | --- | --- |
| *P16-*Met | Forward | TTATTAGAGGGTGGGGCGGATCGC | 150 | 62 |
|  | Reverse | GACCCCGAACCGCGACCGTAA |  |  |
| *P16-*UnMet | Forward | TTATTAGAGGGTGGGGTGGAT TGT | 151 | 60 |
|  | Reverse | CAACCCCAAACCACAACCATA |  |  |
| *TP53*-Met | Forward | GTAGTTTGAACGTTTTTATTTTGG | 115 | 60 |
|  | Reverse | CCTACTACGCCCTCTACAAAC |  |  |
| *TP53*-UnMet | Forward | GTAGTTTGAATGTTTTTATTTTGG | 115 | 58 |
|  | Reverse | CCTACTACACCCTCACAAAC |  |  |
| Ta = annealing temperature | | | | |

**Additional File 2: Figure S1.**

*Normalised Melting Curves and Melting Peaks for P16 Status*

(A) Normalised melt curve; *P16* methylation (B) Derivative melt curve; *P16* methylation


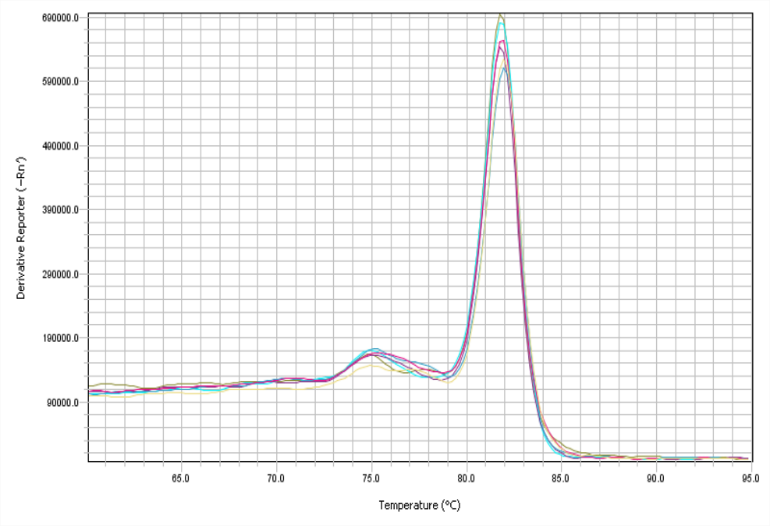

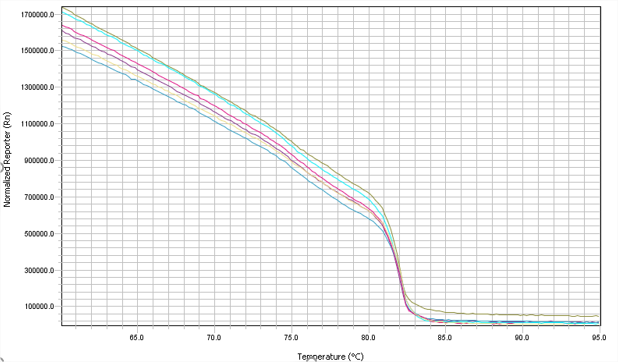


(C) Normalised melt curve; *P16* methylation (D) Derivative melt curve; *P16* methylation


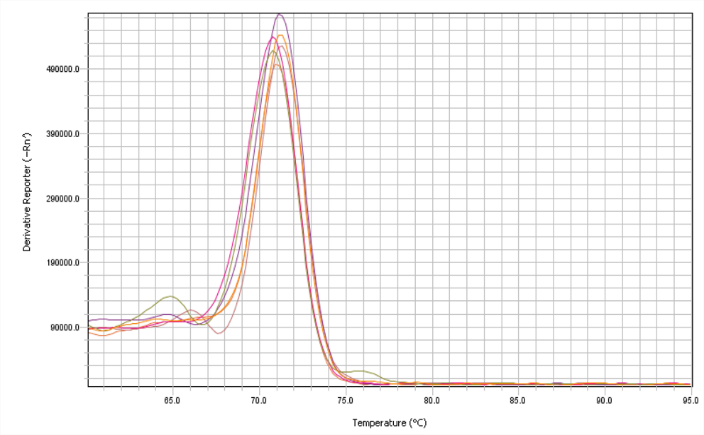

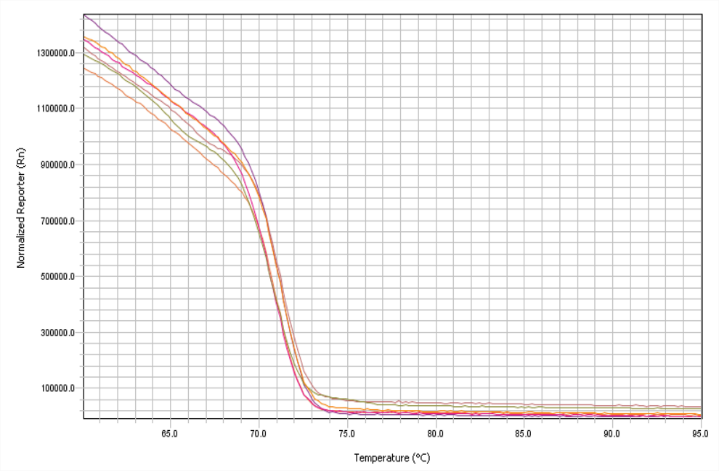


**(A)** Normalised melting curves of *P16* methylated **(B)** Melting peaks of *P16* methylated **(C)** Normalised Melting Curves *P16* unmethylated **(D)** Melting peaks of *P16* unmethylated

**Additional File 3: Figure S2.**

*Normalised Melting Curves and Melting Peaks for TP53 Status*


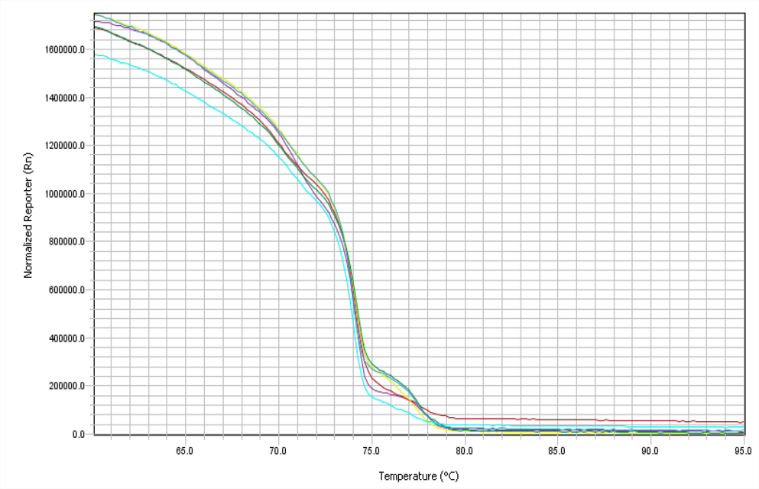
 (A) Normalised melt curve; *TP53* methylation (B) Derivative melt curve; *TP 53* methylation


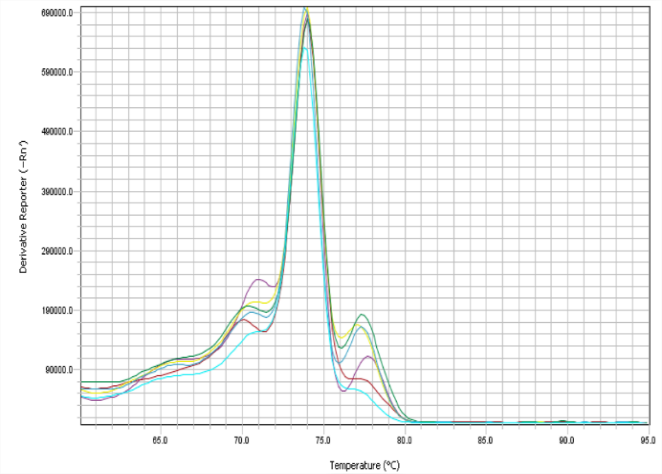


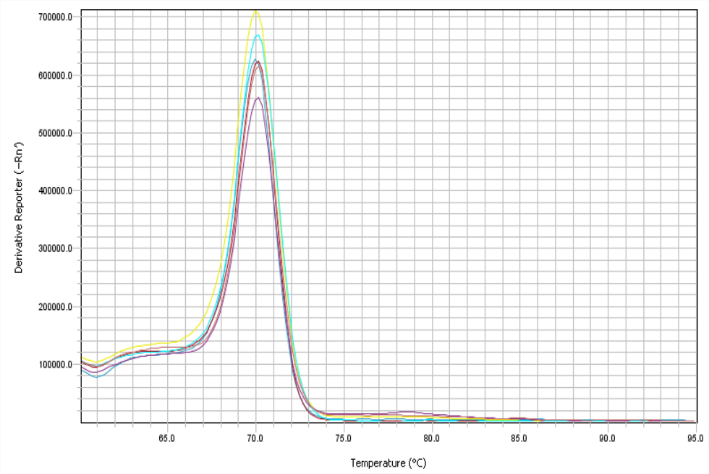

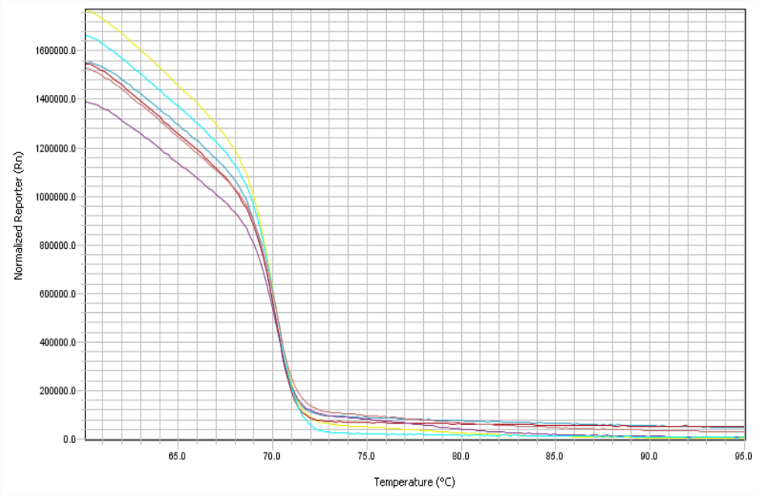
 (C) Normalised melt curve; *TP53* unmethylation (D) Derivative melt curve; *TP53* unmethylation

**(A)** Normalised melting curves of *TP53* methylated **(B)** Melting peaks of *TP53* methylated **(C)** Normalised melting curves *TP53* unmethylated **(D)** Melting peaks of *TP53* unmethylated

**Additional File 4: Table S2**

*Crude and Adjusted Odds Ratios for the Association of Oesophageal Cancer and Environmental Factors*

| **Variables** | **Cases** | | **Control** | | **OR_C_ (95%CI)^a^** | **ORadj (95% CI)^b^** | **P-value** |
| --- | --- | --- | --- | --- | --- | --- | --- |
|  | **N = 105** | | **N = 108** | |  |  |  |
| Gender | | | | | | | 0.103^c^ |
| Female | 42 | (40.0%) | 58 | (53.7%) | 1.00 | 1.00 |  |
| Male | 63 | (60.0%) | 50 | (46.3%) | 1.71 (0.98–2.96)a | 0.19 (0.68–0.57)b |  |
| Age (years) | | | | | | | 0.267^c^ |
| < 60 | 50 | (47.6%) | 60 | (55.5%) | 1.00 | 1.00 |  |
| ≥60 | 55 | (52.4%) | 48 | (44.5%) | 1.35 (0.78–2.33)a | 1.41 (0.76–2.58)b |  |
| Drinking status | | | | | | | 0.002^c^ |
| Non-drinker | 33 | (31.4%) | 65 | (61.0%) | 1.00 | 1.00 |  |
| Drinker | 72 | (68.6%) | 43 | (39.0%) | 3.40 (1.93–6.02)a | 3.44 (1.56–7.68)b |  |
| Smoking status | | | | | | | 0.004^c^ |
| Non-smoker | 37 | (35.5%) | 74 | (68.6%) | 1.00 | 1.00 |  |
| Smoker | 68 | (64.7%) | 34 | (31.4%) | 3.27 (1.86–5.73)a | 3.74 (1.51–8.37)b |  |
| Family history of cancer in first-degree relatives | | | | | | | < 0.001^c^ |
| No | 27 | (25.7%) | 62 | (57.1%) | 1.00 | 1.00 |  |
| Yes | 78 | (74.3%) | 46 | (42.9%) | 3.82 (2.15–6.95)a | 3.56 (1.84–6.91)b |  |
| BMI (kg/m^2^) | | | | | | | 0.903^c^ |
| <23.00 | 49 | (46.7%) | 53 | (51.4%) | 1.00 | 1.00 |  |
| ≥23.00 | 56 | (53.3%) | 55 | (48.6%) | 1.21 (0.71–2.08 )^a^ | 1.03 (0.55–1.95)^b^ |  |
| Marital status | | | | | | | 0.810^c^ |
| Single | 9 | (8.6%) | 9 | (8.3%) | 1.00 | 1.00 |  |
| Married | 88 | (83.8%) | 92 | (85.2%) | 0.98 (0.37–2.61)^a^ | 0.01 (0.35–2.93)^b^ |  |
| Separated | 8 | (7.6%) | 7 | (6.5%) | 1.14 (0.28–4.21)^a^ | 1.21 (0.26–5.63)^b^ |  |
| Betel chewing | | | | | | | |
| Non-betel chewers | 88 | (88.8%) | 93 | (86.1%) | 1.00 | 1.00 | 0.759^c^ |
| Betel chewers | 17 | (16.2%) | 15 | (13.9%) | 1.12 (0.56–2.54)^a^ | 1.14 (0.49–2.63)^b^ |  |

*Note.* ^a^ORc: crude odds ratio, ^b^ORadj.: adjusted odds ratio, 95% CI: 95% confidence interval,

^c^p-value from multivariable logistic regression

| **Environmental Factors** |  | **Methylated *P16*** | **Unmethylated *P16*** | **OR_C_ (95% CI)** | ***P-value*** | **ORadj (95% CI)** | ***P-value*** |
| --- | --- | --- | --- | --- | --- | --- | --- |
| Gender | Female | 53 (43.1%) | 47 (52.2%) | 1.000 | 0.188 | 1.000 | 0.759 |
|  | Male | 70 (56.9%) | 43 (47.8%) | 1.44 (0.84–2.49) |  | 0.87 (0.36–2.07) |  |
| Age (years) | < 60 | 54 (43.8%) | 41 (45.5%) | 1.000 | 0.340 | 1.000 | 0.377 |
|  | ≥60 | 69 (56.2%) | 49 (54.5%) | 1.30 (0.76–2.24) |  | 1.29 (0.73–2.30) |  |
| Drinking status | Non-drinker | 50 (40.6%) | 48 (53.3%) | 1.000 | 0.002 | 1.000 | 0.029 |
|  | drinker | 73 (59.4%) | 42 (46.7%) | 2.38 (1.36–4.15) |  | 2.49 (1.71–3.13) |  |
| Smoking status | Non-smoker | 59 (47.9%) | 57 (63.3%) | 1.000 | <0.001 | 1.000 | 0.003 |
|  | Smoker | 64 (52.1%) | 33 (36.7%) | 2.25 (1.63–5.05) |  | 2.36 (1.36–4.75) |  |
| Family history of cancer | No | 49 (39.8%) | 40 (44.4%) | 1.000 | 0.501 | 1.000 | 0.698 |
|  | Yes | 74 (60.2%) | 50 (55.6%) | 1.21 (0.69–2.09) |  | 1.18 (0.54–1.86) |  |
| BMI (kg/m^2^) | <23.00 | 60 (48.7%) | 43 (47.8%) | 1.000 | 0.885 | 1.000 | 0.808 |
|  | ≥23.00 | 63 (51.3%) | 47 (52.2%) | 1.17 (0.68–2.01) |  | 1.07 (0.59–1.95) |  |
| Marital status | Single | 10 (8.1%) | 8 (8.8%) | 1.000 |  | 1.000 |  |
|  | Married | 102 (82.9%) | 78 (86.6%) | 0.92 (0.35–2.43) | 0.873 | 1.02 (0.38–2.84) | 0.966 |
|  | Separated | 11 (9.0%) | 4 (4.6%) | 1.14 (0.29–4.51) | 0.849 | 1.23 (0.29–5.15) | 0.778 |
| Betel chewing | | | | | | | |
|  | Non-betel chewers | 108 (87.8%) | 73 (81.1%) | 1.000 | 0.180 | 1.000 | 0.273 |
|  | Betel chewers | 15 (12.2 %) | 17 (18.9%) | 0.59 (0.28–1.26) |  | 0.64 (0.29–1.41) |  |

**Additional File 5: Table S3.**

*Associations Between Environmental Factors and P16 Methylation Status*

*Note.* ORc: crude odds ratio, ORadj.: adjusted odds ratio, 95% CI: 95% confidence interval

**Additional File 6: Table S4.**

*Associations Between Environmental Factors and TP53 Methylation Status*

| **Environmental Factors** |  | **Methylated *TP53*** | **Unmethylated *TP53*** | **OR_C_ (95% CI)** | ***P-value*** | **ORadj (95% CI)** | ***P-value*** |
| --- | --- | --- | --- | --- | --- | --- | --- |
| Gender | Female | 51 (43.2%) | 49 (51.6%) | 1.000 | 0.094 | 1.000 | 0.467 |
|  | Male | 67 (56.8%) | 46 (48.4%) | 1.59 (0.92-2.75) |  | 0.73 (0.31-1.72) |  |
| Age (years) | < 60 | 60 (50.8%) | 50 (52.6%) | 1.000 | 0.994 | 1.000 | 0.956 |
|  | ≥60 | 58 (49.2%) | 45 (47.4%) | 0.98 (0.59-1.71) |  | 1.02 (0.62-1.79) |  |
| Drinking status | Non-drinker | 50 (42.3%) | 48 (50.5%) | 1.000 | 0.018 | 1.000 | 0.013 |
|  | drinker | 68 (57.7%) | 47 (49.5%) | 1.94 (1.12-3.69) |  | 2.06 (1.71-3.70) |  |
| Smoking status | Non-smoker | 57 (48.3%) | 59 (62.1%) | 1.000 | 0.045 | 1.000 | 0.041 |
|  | Smoker | 61 (51.7%) | 36 (37.9%) | 1.84 (1.06-3.19) |  | 1.61 (1.15-3.21) |  |
| Family history of cancer | No | 42 (35.6%) | 47 (49.7%) | 1.000 | 0.054 | 1.000 | 0.147 |
|  | Yes | 76 (64.4%) | 48 (50.3%) | 1.77 (0.98-3.07) |  | 1.68 (0.82-4.23) |  |
| BMI (kg/m^2^) | <23.00 | 62 (52.5%) | 41 (43.2%) | 1.000 | 0.337 | 1.000 | 0.229 |
|  | ≥23.00 | 56 (47.5%) | 54 (56.8%) | 0.77 (0.44-1.32) |  | 0.69 (0.39-1.24) |  |
| Marital status | Single | 8 (6.8%) | 10 (10.5%) | 1.000 |  | 1.000 |  |
|  | Married | 101 (85.6%) | 79 (83.2%) | 0.96 (0.36-2.55) | 0.945 | 1.01 (0.37-2.79) | 0.970 |
|  | Separated | 9 (7.6%) | 6 (6.3%) | 0.87 (0.22-3.45) | 0.858 | 0.97 (0.23-4.09) | 0.978 |
| Betel chewing | | | | | | | |
|  | Non-betel chewers | 100 (84.7%) | 81 (85.3%) | 1.000 | 0.916 | 1.000 | 0.851 |
|  | Betel chewers | 18 (15.3 %) | 14 (14.7%) | 1.04 (0.48-2.21) |  | 1.07 (0.49-2.36) |  |

*Note.* ORc: crude odds ratio, ORadj.: adjusted odds ratio, 95% CI: 95% confidence interval

**Additional File 7: Table S5.**

*Interactions Between Environmental Factors and P16 Methylation Status on The Risk of EC*

| **Environmental Factors** |  | ***P16* methylation status** | | | | | | | |
| --- | --- | --- | --- | --- | --- | --- | --- | --- | --- |
|  |  | **Unmethylated *P16*** | |  | **Methylated *P16*** | |  | **Interactions** | |
|  |  | OR_c_ ^a^ (95% CI) | P-value |  | OR_c_ ^a^ (95% CI) | P-value |  | OR_i_ ^a^ (95% CI) | P-value |
| Drinking status | Non-drinker | 1.000 |  |  | 1.17 (0.34-4.04) | 0.805 |  | 1.000 |  |
|  | drinker | 0.58 (0.33-1.01) | 0.056 |  | 2.43 (1.41-4.38) | 0.002 |  | 1.05 (0.31-3.44) | 0.934 |
| Smoking status | Non-smoker | 1.000 |  |  | 0.87 (0.28-2.76) | 0.815 |  | 1.000 |  |
|  | Smoker | 0.53 (0.31-0.94) | 0.030 |  | 2.75 (1.73-5.10) | 0.001 |  | 0.69 (0.21-2.38) | 0.537 |
| Family history of cancer | No | 1.000 |  |  | 0.85 (0.24-2.96) | 0.793 |  | 1.000 |  |
|  | Yes | 0.48 (0.28-0.86) | 0.011 |  | 1.21 (0.69-2.09) | 0.501 |  | 0.35 (0.97-1.29) | 0.115 |
| Betel chewing | Non-betel chewers | 1.000 |  |  | 0.59 (0.71-6.13) | 0.194 |  | 1.000 |  |
|  | Betel chewers | 0.53 (0.30-1.13) | 0.094 |  | 1.41 (0.65-8.09) | 0.187 |  | 0.63 (0.19-2.03) | 0.426 |

*Note.* EC, oesophageal cancer, _c_ combined effect of both environmental factors and methylation status of *P16*.

_i_ interaction between methylation of *P16* and environmental factors. ^a^ adjusted for age, gender, BMI, marital status

**Additional File 8: Table S6.**

*Interactions Between Environmental Factors and TP53 Methylation Status on The Risk of EC*

| **Environmental Factors** |  | ***TP53 methylation status*** | | | | | | | |
| --- | --- | --- | --- | --- | --- | --- | --- | --- | --- |
|  |  | **Methylated *TP53*** | |  | **Unmethylated *TP53*** | |  | **Interactions** | |
|  |  | OR_c_ ^a^ (95% CI) | *P* |  | OR_c_ ^a^ (95% CI) | *P* |  | OR_i_ ^a^ (95% CI) | *P* |
| Drinking status | Non-drinker | 1.000 |  |  | 0.54 (0.14-2.16) | 0.385 |  | 1.000 |  |
|  | drinker | 0.47 (0.26-0.80) | 0.006 |  | 2.07 (1.12-3.37) | 0.018 |  | 1.16 (0.22-2.07) | 0.962 |
| Smoking status | Non-smoker | 1.000 |  |  | 0.51 (0.13-1.98) | 0.326 |  | 1.000 |  |
|  | Smoker | 0.59 (0.33-1.01) | 0.058 |  | 1.84 (1.06-3.19) | 0.029 |  | 1.58 (0.21-2.74) | 0.329 |
| Family history of cancer | No | 1.000 |  |  | 1.23 (0.24-6.33) | 0.802 |  | 1.000 |  |
|  | Yes | 0.44 (0.25-0.78) | 0.005 |  | 1.77 (0.41-3.17) | 0.061 |  | 1.47 (0.16-3.25) | 0.729 |
| Betel chewing | Non-betel chewers | 1.000 |  |  | 0.59 (0.71-6.13) | 0.194 |  | 1.000 |  |
|  | Betel chewers | 0.67 (0.30-1.48) | 0.331 |  | 1.04 (0.48-2.22) | 0.916 |  | 1.25 (1.13-6.81) | 0.846 |

*Note.* EC, oesophageal cancer, _c_ combined effect of both environmental factors and methylation status of *TP53*.

_i_ interaction between methylation status of *TP53* and environmental factors. ^a^ adjusted for age, gender, BMI, marital status

**Additional File 9: Table S7.**

*Effects of Combination and Interaction Between P16 and TP53 Methylation on The Risk of EC*

|  | | **Methylation status** | | | | | |
| --- | --- | --- | --- | --- | --- | --- | --- |
|  |  | **Unmethylated *TP53*** |  | **Methylated *TP53*** |  | **Interactions** | |
|  |  | OR_c_ ^a^  (95% CI) | P -value | OR_c_ ^a^  (95% CI) | P -value | OR_i_ ^a^  (95% CI) | P -value |
| **Methylation status** | **Unmethylated *P16*** | 1.000 |  | 0.45  (0.08-2.46) | 0.359 | 1.000 |  |
|  | **Methylated *P16*** | 0.27  (0.07-1.16) | 0.060 | 13.7  (3.41-5.97) | <0.001 | 4.64  (1.06-8.41) | 0.031 |

*Note.* EC, oesophageal cancer; _c_ combined effect of both environmental factors and methylation status of *P16* and *TP53*. _i_ interaction between methylation status of these genes and environmental factors. ^a^ adjusted for age, gender, drinking, smoking, family history of cancer, BMI, marital status, betel chewing

**Additional File 10: Table S8.**

*Association Between Demographic Characteristics and Prognosis of EC*

| **Demographic Characteristics** | | **Cases (%)** | **HR (95% CI)** | ***P-value*** |
| --- | --- | --- | --- | --- |
| Gender | Female | 42 (40.0) | 1.000 |  |
|  | Male | 63(60.0) | 1.37 (0.87-2.17) | 0.178 |
| Age | <60 | 50 (47.6) | 1.000 |  |
|  | ≥60 | 55 (52.4) | 0.61 (0.38-1.95) | 0.129 |
| BMI (kg/m^2^) | <23.00 | 49 (46.7) | 1.000 |  |
|  | ≥23.00 | 56 (53.3) | 1.22 (0.80-1.87) | 0.351 |
| Family history of cancer | No | 27 (25.7) | 1.000 |  |
|  | Yes | 78 (74.3) | 1.10 (0.64-1.89) | 0.727 |
| Marital status | Single | 9 (8.6) | 1.000 |  |
|  | Married | 88 (83.8) | 0.47 (0.23-1.98) | 0.431 |
|  | Separated | 8 (7.6) | 0.64 (0.31-1.82) | 0.409 |
| Betel chewing | Non-betel chewers | 88 (83.8) | 1.000 |  |
|  | Betel chewers | 17 (16.2) | 1.29 (0.74-2.26) | 0.368 |

*Note.* Oesophageal cancer (95% CI): 95% confidence interval, HR hazard ratio

**Additional File 11: Table S9.**

Multivariate Analysis of Oesophageal Cancer Prognosis

| **Variable** | ***β-coefficient*** | **SE** | ***P*** | **HR (95% CI)** |
| --- | --- | --- | --- | --- |
| Gender | 0.296 | 0.313 | 0.344 | 1.24 (0.72-2.48) |
| Age (years) | 0.614 | 0.239 | 0.036 | 0.54 (0.38-0.86) |
| BMI (kg/m^2^) | 0.039 | 0.321 | 0.904 | 1.03 (0.55–1.95) |
| Metastasis | 0.414 | 0.289 | 0.013 | 1.52 (1.21-2.48) |
| Comorbidity | 0.108 | 0.251 | 0.667 | 1.11 (0.68-1.82) |
| TMN Stage II | 0.243 | 0.509 | 0.604 | 0.74 (0.24-3.01) |
| TMN Stage III (IIIA , IIIB) | 0.741 | 0.468 | 0.046 | 1.25 (1.44-3.42) |
| TMN Stage IV | 0.989 | 0.482 | 0.041 | 2.68 (1.04-6.93) |
